# Dr. Thomas M. Johnson

**Published:** 1916-06

**Authors:** 


**Dr. Thomas M. Johnson,** Buffalo 1861, for most of his life a physician of Buffalo, though at one time interested in a pharmacy, died at Bath, N. Y., May 6, in his 88th year. For many years, he took a prominent part in the Medical Society of the County of Erie. Only 14 members of earlier classes of the University of Buffalo are listed in the Alumni Catalogue as known to be living. Six members of Dr. Johnson's class survive him.

